# The Role of Medicinal and Aromatic Plants against Obesity and Arthritis: A Review

**DOI:** 10.3390/nu14050985

**Published:** 2022-02-25

**Authors:** Alok K. Paul, Rownak Jahan, Anita Paul, Tooba Mahboob, Tohmina A. Bondhon, Khoshnur Jannat, Anamul Hasan, Veeranoot Nissapatorn, Polrat Wilairatana, Maria de Lourdes Pereira, Christophe Wiart, Mohammed Rahmatullah

**Affiliations:** 1 School of Pharmacy and Pharmacology, University of Tasmania, Private Bag 26, Hobart, TAS 7001, Australia; 2 Department of Biotechnology & Genetic Engineering, University of Development Alternative, Lalmatia, Dhaka 1207, Bangladesh; rownak86@hotmail.com (R.J.); afrozebondhon@gmail.com (T.A.B.); jannat.koli.22@gmail.com (K.J.); anamulhasanoris@gmail.com (A.H.); 3 Department of Pharmacy, University of Development Alternative, Dhanmondi, Dhaka 1207, Bangladesh; anita.paul1988@gmail.com; 4 School of Allied Health Sciences, World Union for Herbal Drug Discovery (WUHeDD) and Research Excellence Center for Innovation and Health Products (RECIHP), Walailak University, Nakhon Si Thammarat 80160, Thailand; tooba666@hotmail.com (T.M.); nissapat@gmail.com (V.N.); 5 Department of Clinical Tropical Medicine, Faculty of Tropical Medicine, Mahidol University, Bangkok 10400, Thailand; 6 CICECO-Aveiro Institute of Materials & Department of Medical Sciences, University of Aveiro, 3810-193 Aveiro, Portugal; mlourdespereira@ua.pt; 7 The Institute for Tropical Biology and Conservation, Universiti Malaysia Sabah, Jalan UMS, Kota Kinabalu 88400, Sabah, Malaysia; asianjpharmcog@gmail.com

**Keywords:** rheumatoid arthritis, obesity, spice, medicinal plant, aromatic plant, essential oil, osteoarthritis, comorbidity

## Abstract

Obesity is a significant health concern, as it causes a massive cascade of chronic inflammations and multiple morbidities. Rheumatoid arthritis and osteoarthritis are chronic inflammatory conditions and often manifest as comorbidities of obesity. Adipose tissues serve as a reservoir of energy as well as releasing several inflammatory cytokines (including IL-6, IFN-γ, and TNF-α) that stimulate low-grade chronic inflammatory conditions such as rheumatoid arthritis, osteoarthritis, diabetes, hypertension, cardiovascular disorders, fatty liver disease, oxidative stress, and chronic kidney diseases. Dietary intake, low physical activity, unhealthy lifestyle, smoking, alcohol consumption, and genetic and environmental factors can influence obesity and arthritis. Current arthritis management using modern medicines produces various adverse reactions. Medicinal plants have been a significant part of traditional medicine, and various plants and phytochemicals have shown effectiveness against arthritis and obesity; however, scientifically, this traditional plant-based treatment option needs validation through proper clinical trials and toxicity tests. In addition, essential oils obtained from aromatic plants are being widely used as for complementary therapy (e.g., aromatherapy, smelling, spicing, and consumption with food) against arthritis and obesity; scientific evidence is necessary to support their effectiveness. This review is an attempt to understand the pathophysiological connections between obesity and arthritis, and describes treatment options derived from medicinal, spice, and aromatic plants.

## 1. Introduction

Obesity can be characterized as a body mass index (BMI) of 25 or more in adults, who are classified as overweight, or a BMI of 30 or more, classified as obesity [1]. In 2016, around 1.9 billion adults (aged > 18 years) were overweight, but >650 million people were obese. According to the WHO, the cause of obesity is an increased consumption of foods that are high in fat and sugars, along with the progressively sedentary nature of modern lifestyles, reduced physical work, lack of exercise, and urbanization [1]. An increased waist circumference of more than 40 inches in men (35 inches in women) is known as visceral adiposity, and can be a cause for concern even when BMI is at a normal level. Obesity and overweight also cause other diseases as comorbidities, such as musculoskeletal disorders (e.g., arthritis) [2], cardiovascular diseases [3], diabetes [4], and cancer [5].

### 1.1. Obesity and Inflammation

Obesity is caused by various factors, including imbalance between energy intake and expenditure, sedentary lifestyle, genetics, and many other causes [6]. In terms of cellular mechanisms, adipocytes (cells responsible for the storage of lipids from food and synthesized from de novo lipogenesis) and macrophages secrete adipokines, and excess secretion of adipokines causes low-grade inflammation in some obese people [6,7]. In addition, triglycerides present in adipocytes hydrolyze into free fatty acids, and are transported into the blood circulation of obese people. Lipid deposition in hepatocytes can be seen in disease conditions such as non-alcoholic fatty liver disease (NAFLD) and other comorbidities related to obesity [7]. Heymsfield and Thomas described how obesity is strongly connected to the pathogenesis of several chronic diseases, such as coronary artery disease (CAD), NAFLD, osteoarthritis (OA), gastroesophageal reflux disease, obstructive sleep apnea, stroke, and chronic kidney disease [6]. Immune dysfunction derived from obesity is caused by excess secretion of inflammatory adipokines [8]. A clinical study revealed that obesity was firmly connected with various proinflammatory cytokines, such as interleukins (ILs: IL-5, -10, -12, and -13), interferon-γ (IFN-γ), and tumor necrosis factor-α (TNF-α), and obese patients displayed elevated plasma levels of IL-4, -10, and -13 [9]. Thus, obesity is not simply a result of high energy intake and low energy expenditure; it is multifaceted, and inflammatory cytokines (increased TNF-α, IL-4, and IL-6; reduced IL-10), adipokines (e.g., adiponectin, leptin, resistin, and visfatin), and many other factors are involved in the pathogenesis of obesity [10,11]. The interaction between adipocytes and hepatic lipid metabolism, along with imbalance in the synthesis of de novo synthesis, causes obesity and associated comorbidities [12,13]. Adipocytes release adipokinomes or adipokines that control energy metabolism and dietary intake [14]. Adipokinomes regulate the secretion of adipose cells, releasing fatty acids and prostaglandins, adipsin, proinflammatory cytokines such as IL-1β, -6, -8, and -10, and tumor necrosis factor-α (TNF-α) [10,15,16]. Excess plasma IL-6 levels trigger the release of C-reactive proteins by hepatocytes, which indicate the levels of chronic inflammation and the risk of cardiovascular disorders [17]. Collectively, these processes lead to lipid deposition (obesity), vascular hemostasis, insulin resistance, chronic metabolic diseases such as type 2 diabetes, and inflammation, as the proinflammatory cytokines transform into inflammatory cytokines [17,18,19]. Inflammatory processes also stimulate the development or progression of psoriasis, cancer, and kidney diseases [17]. Increased plasma contents of IL-6, IL-10, and IL-18 are observed among obese patients [20,21]. Thus, obesity is not merely a metabolic disease; rather, it is a chronic inflammatory disorder, where dietary intake inflicts or triggers the pathogenesis of obesity and diabetes [11,22].

### 1.2. Influence of Dietary Habits during Childhood on Obesity and Inflammation

**Figure 1:**
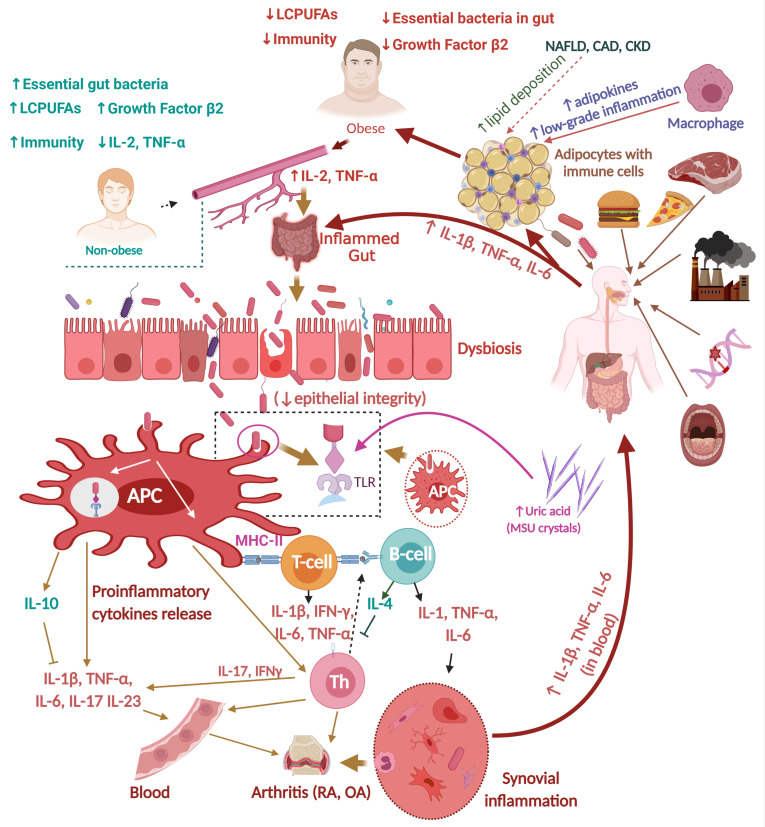
Mechanisms of obesity and rheumatoid arthritis (RA). Abbreviations—APC: antigen-presenting cell; GI: gastrointestinal: GIT: gastrointestinal tract; IL: interleukin; LCPUFAs: long-chain polyunsaturated fatty acids: TNF-α: tumor necrosis factor alpha; IFN-γ: interferon gamma; M-cell: microfold cell; Th: T helper cell; T-cell: T-cell lymphocytes: B-cell: B-cell lymphocytes; red rod-shaped bacteria: *Prevotella* spp.; SCFA: short-chain fatty acid. This figure was made with www.biorender.com (accessed on 25 January 2022).

As mentioned in the previous section, there are possible correlations between obesity and adult diet. Similarly, childhood diet may influence the possibility of obesity and other comorbidities in later life. Breastfeeding (intake of colostrum and milk) at an earlier age (from birth to 6 months of age) reduces plasma proinflammatory cytokine levels compared to formula feeding [23] (Figure 1). Breast milk is an ideal food for children, naturally supplemented with various bioactive immunomodulatory substances such as immunoglobulins (e.g., secretory IgA), oligosaccharides, cytokines (e.g., IL-1, IFN-α, IL-6, and TNF-α help in the development and functions of the mammary gland), growth factors (e.g., transforming growth factor β_2_), food antigens, and essential microbiota supplements (e.g., non-sterile breast milk contains long-chain polyunsaturated fatty acids (LCPUFAs), which impede the production of proinflammatory cytokines [24,25,26] (Figure 1). Human milk intake in infancy can also protect children against various pathogens, including but not limited to *Bordetella pertussis, Campylobacter, Haemophilus, Salmonella, Streptococcus, Shigella, Vibrio cholerae*, and respiratory viruses [23,27,28]. Noticeably, formula-fed children show reduced transforming growth factor β_2_ compared to breast milk-fed children; instead, they display higher levels of plasma proinflammatory cytokines (e.g., IL-2 and TNF-α) than breast-fed children [29]. Inadequate intake of LCPUFAs in the body/diet can influence the development of obesity and arthritis (Figure 1). Resistin—an adipokine—along with other senescence-associated secretory phenotype factors, regulates glucose metabolism, oxidative stress, inflammatory responses, and autoimmune diseases. [30,31]. High plasma resistin concentrations can increase the possibility of inflammation, insulin resistance, and the aging process [30]. Resistin possibly interacts with TLR-4 receptors, influences the transcription of proinflammatory genes, inflammatory cytokines, and chemokines, and causes osteoclastogenesis via the NF-κB pathway [30]. Sedentary lifestyle and increased calorie intake are related to the progression of adipocyte hypertrophy and low-grade inflammation via the recruitment of antigen-producing cells in adipose tissues [31,32]. Resistin, adiponectin, TNF-α (released by adipocytes), and proinflammatory cytokines (e.g., IL-1β, IL-6) derived from adipokines increase muscle and bone metabolism [30,32]. This biological pathway is responsible for the generation of several chronic diseases, including obesity, diabetes, and arthritis [32].

## 2. Arthritis and Inflammation

### 2.1. Osteoarthritis and Inflammation

**Figure 2:**
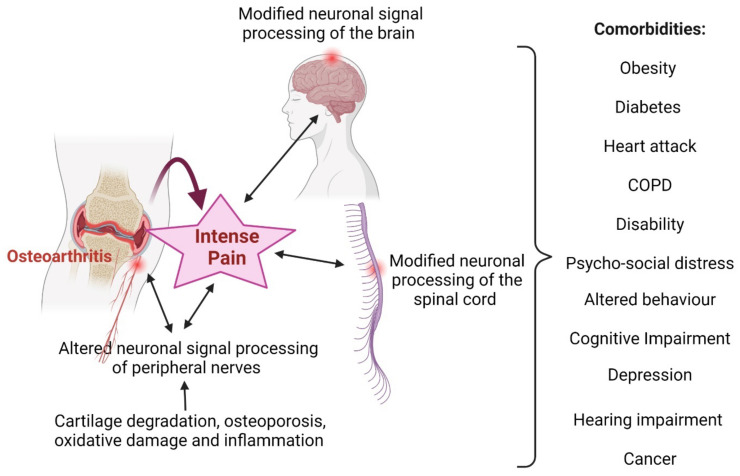
Osteoarthritis (OA) and associated comorbidities. Abbreviations—COPD: chronic obstructive pulmonary disease. This figure was made with www.biorender.com (accessed on 25 January 2022).

There are various types of arthritis, and these are multifactorial, with the common features of chronic intense pain and inflammation [33]. Osteoarthritis (OA) is a chronic painful disorder that increases with age and is common in adults aged over 55 years [34]. The mechanisms of OA are not completely understood, but its clinical features include irreversible age-related damage to the joint cartilage, pain, and low-grade inflammation over a period of many years [35]. The pathogenesis of OA can also be caused by cellular stress produced by the activation of endogenous cytosolic proteins such as nucleotide-binding domain, leucine-rich repeat/pyrin domain-containing-3 (NALP3) inflammasome [36,37,38,39,40], proinflammatory cytokines released by macrophages [41,42,43], or the production of proinflammatory cytokines induced by uric-acid-crystal-induced inflammasome assembly [40]. There is a positive correlation between osteoarthritis (OA) severity, uric acid levels (in synovial fluid), and proinflammatory cytokines (e.g., IL-18, IL-1β) [38,44]. Monosodium uric acid (MSU) can accumulate in joints as crystals when its plasma concentrations exceed its solubility (≥70 mg/L) [45], stimulating the synthesis of different inflammatory cytokines [46]. The inflammatory processes are also triggered by chemokines, proteases, and oxidative materials that cause osteoporosis, cartilage degradation, and inflammation in the synovial joints [44,47]. This process is further exaggerated when toll-like receptors recognize MSU (monosodium urate crystals), and when lymphocytes and macrophages in synovial fluids uptake MSU. These interactions ultimately release various inflammatory cytokines (especially IL-1β, IL-6, TNF-α, and IL-18) via nucleotide-binding domain and the leucine-rich repeat/pyrin domain-containing-3 (NALP3) inflammasome [36,37,38,39,40]. OA is also influenced by calcium-oxalate-containing crystals that stimulate the production of IL-1β, causing cartilage damage [48]. OA is also caused by mutations of genes encoding collagens (e.g., types II, IV, V, and VI) [33,49]. The pathogenesis of OA can cause neuronal damage in joint tissues, causing intense pain, limited mobility, depression, and anxiety in elderly people (Figure 2) [50,51]. People experiencing OA also often have multiple comorbidities, such as obesity [52,53,54], diabetes [4], cardiovascular diseases [3], cancers [5], and musculoskeletal disorders [2].

**Figure 3:**
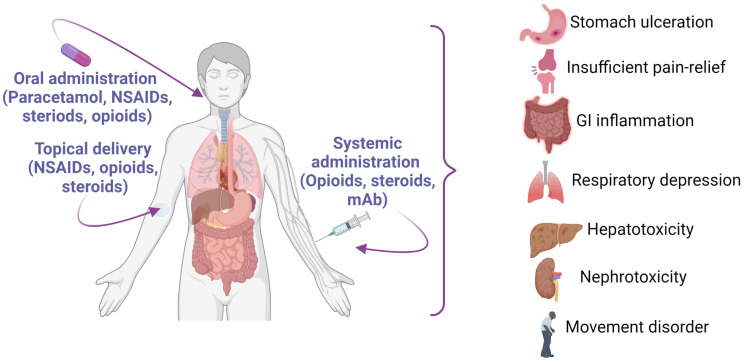
Routes of administration of commonly used drugs for the treatment of OA, and some adverse effects associated with these drugs. Abbreviations—NSAIDs: non-steroidal anti-inflammatory drugs (NSAIDs); mAb: monoclonal antibody. This figure was made with www.biorender.com (accessed on 25 January 2022).

Currently, analgesics such as non-steroidal anti-inflammatory drugs (NSAIDs) and corticosteroids are used to manage OA, but these drugs have no effect on the prevention of OA’s pathogenesis, and they are mainly for symptomatic management. In addition, these drugs have adverse effects on the gut, liver, kidneys, and heart [55,56,57,58]. Long-term use of NSAIDs to manage arthritis provides poor pain relief, major discomfort for patients, and can lead to invasive procedures, such as surgeries [59,60,61] (Figure 3). A clinical study showed that paracetamol alone provided insufficient analgesia in OA, but an NSAID such as diclofenac (0.15 g daily) showed noticeable efficacy in OA management [62]. It is also important to note the adverse effects of diclofenac, including gastrointestinal toxicity, liver toxicity, and renal impairment (Figure 3). Another study reported that celecoxib (an NSAID) caused lower cardiovascular, renal, and gastrointestinal adverse reactions than ibuprofen or naproxen (NSAIDs), which was similar in patients experiencing OA or RA [63]. It is understood that there are significant variations between drugs within a group of drugs (e.g., NSAIDs), and the efficacy of a particular drug may depend on its molecular structure, formulation, route of administration, dosage, and duration of treatment [64]. The bioactivity and efficacy of a drug also depend on its metabolic capabilities (e.g., hepatic or renal impairment, aging-related) and bioavailability at the target site. Importantly, chronic treatment with NSAIDs for OA can result in adverse outcomes and cause adverse events in older adults [65,66]. Opioids are not recommended to manage OA [51,67], but these drugs are widely used in OA-related chronic pain management for older adults, despite their potential adverse effects—such as addiction, dependence, analgesic tolerance, respiratory depression, and behavioral disorders over long-term usages (Figure 3) [66,68,69,70,71].

### 2.2. Brief Pathophysiology of Rheumatoid Arthritis (RA)

#### 2.2.1. RA and Inflammation

Despite differences in the initiation and progression mechanisms between OA and RA—the latter of which is another type of arthritis that is multifactorial, and whose root causes remain to be elucidated—long-term low-grade inflammation is the common ground in the pathogenesis of obesity, OA, and RA [52,53,54,72]. As with the pathogenesis of OA, RA manifests with increased secretion of proinflammatory cytokines (e.g., IL-1, IL-6, IL-12, IL-17, IL-18, and TNF-α). In parallel, secretion of immunomodulatory cytokines (e.g., IL-10, IL-11, and IL-13) is reduced in the blood, along with stiffness, swollen joints, and impaired movement of the affected person [73,74,75,76] (Figure 1). Defensive cells, such as T helper 1 (Th1) and T helper 17 (Th17) cells, produce an inflammatory response via IL-17A, IFN-γ, and TNF-α, leading to the pathogenesis of RA [77,78]. Toll-like receptors (TLRs) regulate the functions of the nuclear factor kappa B ligand (NF-κB), osteoclastogenesis, and generation of proinflammatory cytokines [79,80,81]. As a result, joint pain, inflamed joints, and damage to cartilage can be seen during clinical symptoms of RA [82]. Inflammatory cytokines such as IL-17 or TNF-α can influence the upregulation of matrix metalloproteinase (MMP) enzymes, which irreversibly damage the extracellular matrix and the cartilage of joints [74,83] (Figure 1). Apart from inflammatory or genetic mechanisms, fat-rich food intake, smoking, and periodontal infections also affect the generation and progression of RA [84,85]. Women are more prone to RA than men. Citrullination of proteins in lung macrophages, along with neuropathic pain and osteoporosis, can potentially influence the pathogenesis of RA [84,86,87,88].

#### 2.2.2. RA, Gut Dysbiosis, and Inflammation

RA also manifests as a result of excess inflammatory cytokines, with the influence of major changes in the microbial population of the gut. For example, *Faecalibacterium* spp. are a part of the healthy gut microbiota that is responsible for butyrate production [89,90,91], and helps in the secretion of mucin—a natural lubricator of gut epithelial cells. If the abundance of *Faecalibacterium* spp. decreases, other opportunistic bacteria such as *Collinsella, Eggerthella, Haemophilus, Prevotella*, and *Streptococcus* can grow and produce inflammatory cytokines and/or cause citrullination of proteins, leading to RA [90,92].

*Prevotella copri* (*P. copri*) is a part of our normal gut microbiota and oral cavity, and can grow massively with the influence of change in diet, stress, lack of oral hygiene, and microbial infection [85,93,94,95]. As a result, *P. copri* can cause increased production of T helper cells (e.g., Th1, Th17) and inflammatory cytokines (e.g., IL-1β, IL-6, IL-17, and IL-23), leading to an inflammatory response in the gut, and can possibly migrate to inflammatory joint tissues [96,97] (Figure 1). *Prevotella* spp. can produce increased prostaglandin E2 in joint tissues, and has been observed in RA, causing joint pain, inflammation, and bone degradation [98,99] (Figure 1). The simultaneous growth of *Porphyromonas gingivalis* in the mouth and *P. copri* in the intestine are noticed in RA patients [100]. *P. gingivalis* possibly translocates to synovial joints via phagocytosis, causes citrullination of proteins in joints, and increases inflammatory cytokine production [101,102]. Proper management and restoration of healthy gut microbiota by using probiotic supplements as food can reduce the population of *Prevotella* spp. and increase the gut population of *Lactobacillus* spp. [95].

## 3. Relationships between Obesity and Arthritis

**Figure 4:**
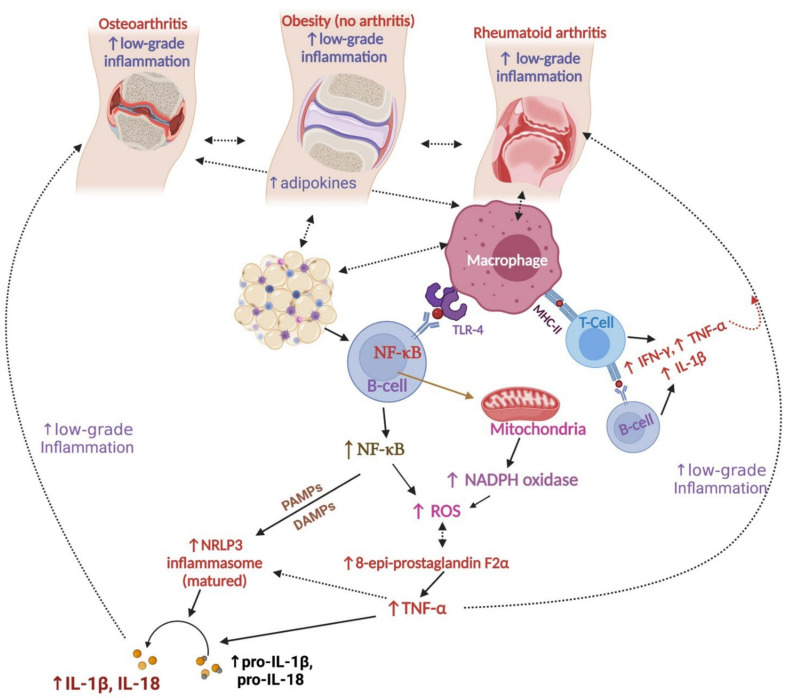
Relationships between the pathogenesis of osteoarthritis (OA), obesity, and rheumatoid arthritis (RA) in older adults. Abbreviations—↑: increase; ROS: reactive oxygen species; TNF-α: tumor necrosis factor α: TLR: toll-like receptor; IL: interleukin; NADPH: nicotinamide adenine dinucleotide phosphate oxidase; IFNγ: interferon gamma; NF-κB: nuclear factor kappa B; NLRP3: nucleotide-binding oligomerization domain-like receptor family pyrin domain-containing 3 inflammasome; PAMPs: pathogen-associated molecular patterns; DAMPs: damage-associated molecular patterns; MHC-II: major histocompatibility complex class II. This figure was made with www.biorender.com (accessed on 25 January 2022), and partially reproduced from Paul et al. [107].

OA and RA are both prevalent in older adults (>55 years), and especially in the elderly with frailty syndromes (e.g., falls, immobility, delirium, incontinence, and adverse effects of medications) [72,103]. Obesity is also a common comorbidity of this population cohort for various reasons, including inactivity, diet, diabetes, and aging [104,105]. Tumor necrosis factor α (TNF-α)—a proinflammatory cytokine—from the adipose tissues of obese animals can cause low-grade inflammation in adipocytes [52,106]. Adipose tissues mainly produce inflammatory biomarkers such as TNF-α, and macrophages and other immune cells are partially responsible for oxidative damage and low-grade inflammation in the body [52,106,107]. NLRP3 (nucleotide-binding oligomerization domain-like receptor family pyrin domain-containing 3)—a polyprotein complex inflammasome found in macrophages—is also responsible for releasing proinflammatory cytokines. NLRP3 is stimulated by the activation of NF-κB (nuclear factor kappa B, which TNF-α stimulates), and causes the secretion of the proinflammatory cytokines pro-IL-1β and pro-IL-18 [108] (Figure 4). NLRP3 is matured by PAMPs (pathogen-associated molecular patterns) and DAMPs (damage-associated molecular patterns) or lipopolysaccharides. NLRP3 maturation stimulates the release of cytokines (e.g., IL (interleukin)-1β, IL-6, and IL-18) and low-grade inflammation in multiple organs, including joints (Figure 4) [109].

Clinical studies show strong positive connections between obesity, osteoarthritis, and rheumatoid arthritis [52,53,110,111,112] (Figure 4). People with a body mass index of >30 kg/m^2^ show higher incidence of knee OA than people of normal weight, and it is recommended to reduce weight in order to improve clinical symptoms of OA in obese patients [113,114]. A clinical study showed that obesity was present (33.4%) in RA patients (*n =* 11,406) at a significantly higher rate than obesity (31.6%) in the control group (*n =* 54,701) [112]. Obesity causes inflammation and autoimmune conditions in RA patients [112]. Obese RA patients experience more tender joints and swelling in joints than non-obese RA patients [115]. Obesity is a common comorbidity of RA patients, and it also reduces the efficacy of drugs working against TNF-α, but losing body weight improves the success of treatment with these drugs [111]. Importantly, no association with BMI was found in this review with drugs other than anti-TNF-α drugs, such as biologics that act against IL-6, CD4, or CD20 [111]. Studies have reported that the RA patients experience lower grip strength and fatigue (40–80%), and these decrease their strength and their interest in being involved in various physical activities [116,117]. Similarly, a later study showed that patients who also experienced RA displayed fatigue (40%) and anxiety/depression (52%) as comorbidities [118]. Obese RA patients experienced less remission (improvement of symptoms and pain relief) and lower disease activity scores than non-obese (control) RA patients [110]. Van Beers-Tas et al. mentioned that reduced smoking increased arthritis remission, but obesity increased arthritis progression and delayed its remission [119]. Another study on a small number (*n =* 19) of obese RA patients (aged 55 years on average; range: 34–71) observed that reduction in dietary energy intake and moderate physical exercise led to a 9% reduction in fat mass and improved physical fitness of the participants [120]. Conversely, another study with a comparatively large number (*n* = 192) of participants (aged 64.5 years on average, range: 50–78) in a similar weight-reduction program did not improve structural joint damage, muscle strength, or knee joint alignment, but achieved some benefits in terms of overall health improvement [121]. Noticeably, age was an important factor in the performance of the participants, and there were differences in the measurements of performance, as the previous study measured outcomes such as the capability to ride a bicycle, whereas the later study investigated using MRI and radiographs [120,121]. Collectively, management of obesity may improve the clinical symptoms of obese OA and RA patients.

## 4. Current Drugs for the Management of Obesity and Arthritis

A few anti-obesity drugs have now been approved for human use, and most of these show various side effects. According to the National Institute of Diabetes and Digestive and Kidney Diseases (NIDDK), the United States Food and Drug Administration (USFDA) has so far approved five drugs—namely, orlistat, phentermine/topiramate, lorcaserin, naltrexone/bupropion, and liraglutide—to treat obesity [122]. Importantly, the European Medicines Agency (EMA) has approved three drugs to fight symptoms of obesity: orlistat, bupropion/naltrexone, and liraglutide [123]. Orlistat reduces intestinal absorption of fat content from food, as it is a pancreatic lipase inhibitor; side effects of this drug include diarrhea, oily stools, abdominal pain and, less frequently, cholelithiasis, cholestatic hepatitis, and subacute hepatitis [124]. People feel less hungry when using the drug combination phentermine/topiramate, as phentermine decreases one’s appetite, while topiramate reduces seizures and migraine headaches. Noticeably, this drug combination can cause serious side effects, including dysgeusia (taste alteration), paresthesia (burning sensation in hands and feet), hypoesthesia (loss of sensation of a body part), attention deficiency, dizziness, constipation, and dry mouth [125]. There are serious safety concerns with respect to the long-term efficacy of anti-obesity medications; the European Medicines Agency refused the approval of phentermine/topiramate, while for lorcaserin, authorization was previously withdrawn for a low overall benefit/risk ratio [126]. Lorcaserin (for the risk of cancer), rimonabant, and sibutramine have been withdrawn from the US market for safety concerns [126,127]. Mitral regurgitation is a serious side effect of lorcaserin, and may lead to other complications, such as increased risk of cardiovascular complications [127,128]. The naltrexone/bupropion drug combination has little effect against obesity individually. Long-term opioid treatment causes various behavioral adverse effects, addiction, and tolerance, but naltrexone—as an opioid antagonist—shows efficacy against dependency on opioids and alcoholic beverages [64,69,122,129]. Patient management using these analgesics should also consider the reduced metabolic capability of people such as the elderly, or people suffering from chronic kidney or liver diseases [70,130,131,132,133]. Bupropion is used for treating depression and for help with giving up smoking. Individually, these drugs have no or little effect on obesity; used in combination, they form a safe anti-obesity polypharmacy drug with no serious side effects except for nausea [134,135].

Liraglutide—an anti-diabetic drug—works as an anti-obesity drug as well, and shows side-effects such as nausea, diarrhea, abdominal pain, and constipation. Acute pancreatitis and rare thyroid tumors are severe adverse effects that may arise from the use of liraglutide [136].

**Figure 5:**
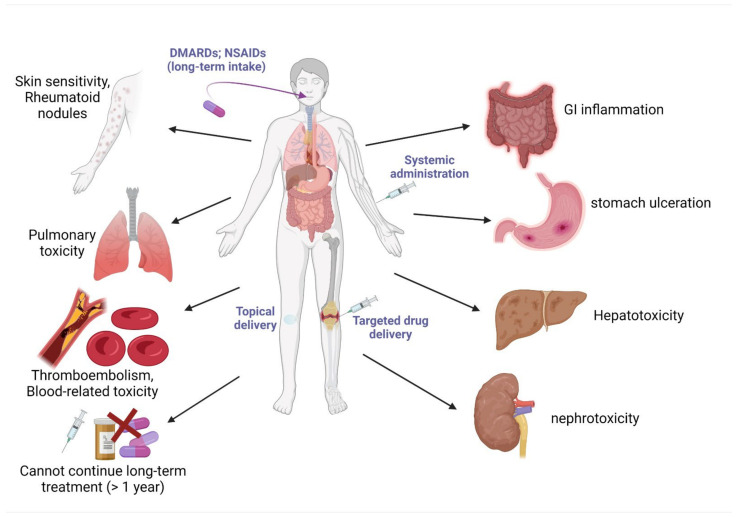
Common problems associated with long-term treatment of arthritis with current anti-arthritic drugs. Abbreviations—NSAIDs: non-steroidal anti-inflammatory drugs; DMARDs: disease-modifying anti-rheumatic drugs; GI: gastrointestinal. The purple-colored text indicates common routes of administration of various anti-arthritic drugs. This figure was made with www.biorender.com (accessed on 25 January 2022).

Rheumatoid arthritis (RA) is currently treated with disease-modifying anti-rheumatic drugs (DMARDs) such as methotrexate, non-steroidal anti-inflammatory drugs (such as paracetamol, ibuprofen, naproxen, diclofenac, indomethacin, ketoprofen, and meloxicam), Janus kinase (JAK) inhibitors (e.g., baricitinib and upadacitinib), anti-malarial drugs (e.g., hydroxychloroquine and chloroquine), TNF-α inhibitors, and glucocorticoids (e.g., prednisone, hydrocortisone, and dexamethasone). All of these drug types produce severe adverse effects (Figure 5), limiting their efficacy, and scientists are looking for safe alternative drugs or food supplements for the prevention or cure of RA [95,137,138,139].

Overall, anti-obesity drugs are effective in reducing body weight, but in consideration of their adverse effect profiles, the only possible alternative to these drugs is bariatric surgery, which also increases the risk of developing alcohol use disorders [140]. Thus, scientists, naturopaths, and traditional medicinal practitioners are investigating some suitable plants that have the ability to reduce weight. A single plant may contain hundreds of secondary metabolites, a few of which may be effective against obesity. Plants are readily available from nature; many plants can be cultivated and extracted to isolate active ingredients for various purposes.

## 5. Research Methodology

To select the information on medicinal or aromatic plants for use against obesity and rheumatoid arthritis (RA) for this review, data from recent literature (no time limit used) were gathered from the PubMed, Scopus, and Google Scholar databases. The keywords used for the literature research included the terms obesity, anti-obesity, rheumatoid arthritis, RA, medicinal plant, essential oils, and preclinical and clinical studies.

## 6. Obesity and Arthritis Management

### 6.1. Ayurvedic Medicines against Arthritis and Obesity

Ayurvedic medications have been used on the Indian subcontinent since the 2nd century BC, and are still being used as traditional, complementary, and alternative medicines [141]. There are many Ayurvedic plants and drug formulations that are used to manage arthritis and inflammatory diseases [142,143,144,145,146]. Recent randomized clinical trials (RCTs) of several Ayurvedic drugs (e.g., Rumalaya (*Moringa oleifera*; *Tinospora cordifolia*), Shunti-Guduchi (*Zingiber officinale*; *Tinospora cordifolia*), Ashwagandha powder (*Withania somnifera*), and Sidh Makardhwaj (gold, mercury, and sulfur in a specific ratio of 1:8:24, and prepared according to the Ayurvedic Formulary of India [147]) reported efficacy against osteo- and rheumatoid arthritis [148,149,150]. It is to be noted that despite the presence of mercury in Sidh Makardhwaj, it has been claimed that the formulation has no detectable toxic effects [147]. The following plants are mainly used as medications against arthritis in Ayurveda: *Curcuma longa* L., *Boswellia serrata* Roxb. ex Colebr., *Zingiber officinale* Roscoe, *Tinospora cordifolia* (Willd.) Miers, *Withania somnifera* (L.) Dunal, *Commiphora myrrha* (Nees) Engl., *Glycyrrhiza glabra* L., *Piper nigrum* L., and *Capsicum spp.* (Table 1). *Curcuma longa* has shown anti-inflammatory and anti-arthritic effects in various clinical and preclinical studies. The rhizome of *Curcuma longa* is traditionally used as a spice in Indian cuisine and for medicines in Ayurveda. The rhizome of this plant is known to be effective against asthma, allergies, rheumatism, liver disorders, and inflammation in Ayurvedic medicines. A recent clinical trial demonstrated that 0.5 g twice daily consumption of *Curcuma longa* extract (composition: 80% wt/wt aqueous-based extract standardized to turmerosaccharides, and 20% wt/wt curcuminoids) over 12-week period improved symptoms (such as knee pain using both the visual analogue scale (VAS) and Western Ontario and McMaster Universities Osteoarthritis Index (WOMAC) pain values) of patients experiencing symptomatic knee osteoarthritis and knee effusion synovitis [151]. 

*Boswellia serrata* is known as an Ayurvedic medicine used against rheumatic pain and inflammatory diseases. In recent years, several randomized clinical trials found that the extracts of *Boswellia serrata* provided relief from arthritis-related pain and stiffness from knee osteoarthritis [155], reduced inflammatory cytokines, and improved Western Ontario and McMaster Universities Osteoarthritis Index and visual analog scale scores [157]. Supplementation of 100 mg of *Boswellia serrata* extract with 300 mg of hyaluronic acid (1 tab/day for 20 days) improved arthritis-pain-related visual analogue scales (e.g., the American Knee Society Score (AKSS) and visual analogue scale (VAS) for pain) [167]. *Curcuma longa* (350 mg extract) and *Boswellia serrata* extract (150 mg) twice daily for 12 weeks also improved OA pain in patients with moderate knee OA using the Western Ontario and McMaster Universities Osteoarthritis (WOMAC) Index and visual analogue scale (VAS) [168].

**Figure 6:**
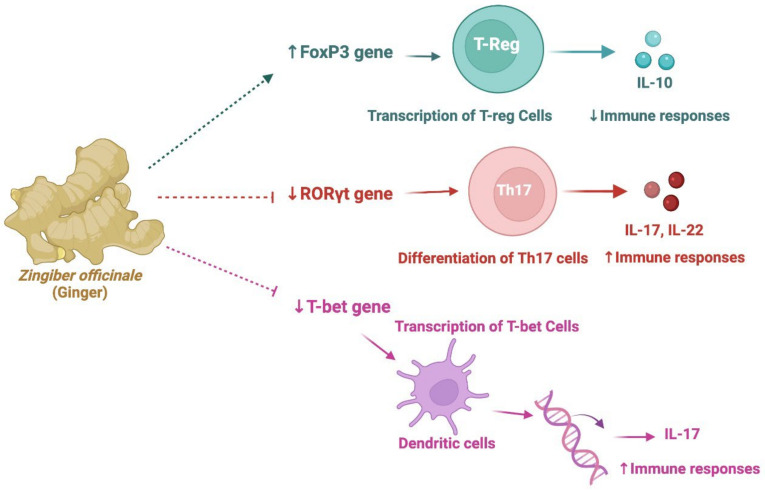
Simplified mechanisms of the immunomodulatory effects of *Zingiber officinale*. Abbreviations—T-reg cells: regulatory T-lymphocytes; Th17: helper T-lymphocyte 17; IL: interleukin; FoxP3: forkhead-box-P3; RORγt: retinoic-acid-receptor-related orphan nuclear receptor gamma. This figure was made with www.biorender.com (accessed on 10 January 2022).

Ginger (rhizomes of *Zingiber officinale*) improved RA by increasing expression of the forkhead-box-P3 (FoxP3) gene, and by reducing the expression of retinoic-acid-receptor-related orphan nuclear receptor gamma (RORγt) and T-bet genes [165] (Figure 6). FoxP3 is an essential transcription factor of regulatory T (T-reg) cells, and expression of this factor helps the development and function of T-reg cells [169]. Activation of T-reg cells produces the immunomodulatory cytokines transforming growth factor (TGF)-β and interleukin-10 (IL-10), and reduces inflammation [169]. The transcription factor RORγt is considered to be a major regulator of the differentiation of T helper 17 cells (Th17 cells) and the production of IL-17 family cytokines, which play an essential role in the development of a number of autoimmune disorders, including arthritis. T-bet is an immune cell transcription factor; in dendritic cells, T-bet reportedly regulates the production of the proinflammatory cytokine IL-1α [170]. Cumulatively, the decrease in the expression of these two transcription factor genes can be helpful in ameliorating RA (Figure 6) [171]. In another study with knee OA patients, 12 weeks of treatment with ginger (750 mg capsule) and ginger supplemented with diclofenac (750 mg capsule with a 50 mg diclofenac tablet) improved OA [163]. Noticeably, no severe adverse effects were recorded in these studies. Importantly, a *Tinospora cordifolia*-*, Zingiber officinale*-, and *Semecarpus anacardium*-containing Ayurvedic drug reportedly improved the symptoms of OA [158]. Furthermore, another clinical study over a period of 24 weeks with co-treatment with an Ayurvedic formulation containing mixed extracts of *Tinospora cordifolia, Boswellia serrata, Emblica officinalis,* and *Zingiber officinale*, along with glucosamine sulphate (2 g/day) and celecoxib (0.2 g/day), reduced symptoms of knee OA [160]. A three-month treatment with a herbal formulation containing mixtures of powders of *Withania somnifera* (roots), *Boswellia serrata* (stem), *Curcuma longa* (rhizomes), and zinc produced better pain relief and reduced the disability scores of patients with knee OA [172].

*Commiphora* species of plants have been used in traditional medicine as painkillers and anti-inflammatory agents. In a recent clinical trial, a TCM medicinal formulation containing extracts of *Commiphora myrrha* (gum resin) and *Paeonia lactiflora* (root) showed pain relief and no severe adverse effects when given over a period of 12 weeks in people experiencing knee OA [161].

*Piper nigrum* is traditionally used in many Ayurvedic formulations [166]. The main alkaloid of fruits of this plant is piperine. Daily treatment twice for 4 weeks with herbal capsules containing curcumin (300 mg), gingerols (7.5 mg), and piperine (3.75 mg) reduced the prostaglandin E2 levels in people experiencing chronic knee OA [166]. Another plant—*Typhonium trilobatum* (L.) Schott (Ghatkul, Ghetkun)—is known to have anti-arthritic and anti-rheumatic effects (leaf and whole plant, respectively). A preclinical study confirmed that the plant showed anti-inflammatory and analgesic effects, but the study needs further evidence from actual clinical trials [173,174]. It can be concluded that various trials have shown the efficacy of a number of Ayurvedic and other traditional medicinal formulations in the treatment of arthritis; however, more clinical trials are necessary, as there are various discrepancies between the settings of clinical trials, such as the population sizes, types of patients, methodologies, and duration of treatment. Using modern formulations, such as implementation of nanotechnology-based formulations, would increase the bioavailability of some of these phytochemicals (such as curcumin), as shown in experimental studies [175,176].

In addition to the anti-arthritic plants mentioned above, Mukhopadhyay et al. (2019), in their list of anti-arthritic plants, mentioned *Cuscuta reflexa* Roxb., *Piper longum* L., *Coriandrum sativum* L., *Cinnamomum zeylanicum* Blume, *Caesalpinia pulcherrima* (L.) Sw., *Asparagus racemosus* Willd., *Abutilon hirtum* (Lam.) Sweet, *Terminalia pallida* Brandis, *Lawsonia inermis* L., *Trigonella foenum-graecum* L., *Punica granatum* L., *Ruta graveolens* L., *Terminalia chebula* Retz., *Sida rhombifolia* L., *Xanthium strumarium* L., *Vitex negundo* L., *Lantana camara* L., and *Citrullus colocynthis* (L.) Schrad. for their uses in the alleviation of arthritis [177,178,179]; their phytoconstituents and other details are shown in Table 2.

### 6.2. Essential Oils for Use against Arthritis and Obesity

Apart from the intake of traditional medicines, massages and complementary therapies using essential oils are also claimed by traditional medicinal practitioners (TMPs) to improve the symptoms of various diseases—especially from arthritis or chronic pain. These beliefs stem from the practices and customs learned in various human societies over hundreds or thousands of years, and oral passage of the knowledge gained from generation to generation before the arrival of writing and record keeping on clay tablets, papyrus, or paper [353]. Essential oils are volatile aromatic oils isolated from flowers, barks, leaves, and other parts of specific plants. Many of these oils have antimicrobial, emollient, palatable, and lipophilic permeability through the skin. Essential oils give people a good feeling at spiritual, physical (via massaging), and olfactory levels. The efficacy of these oils against chronic arthritic pain is yet to be established. Some preliminary clinical trials with a few essential oils have shown some benefits against arthritis, but their efficacy over a long period of time is unknown (Table 3). In our search of the PubMed search engine, we found four randomized clinical trials (Table 3) of essential oil therapy. Out of these four trials, aromatherapy with essential oils was used in two studies, whereas oral or gargling administrations were used in the other two trials [354,355,356,357]. A recent systematic review reported that essential oils have been used to treat RA, mainly with a small number of subjects for a short duration of observation (2–12 weeks), and mostly with women (60–100%) [358]. The typical oil used for aromatherapy to treat RA was lavender, ginger, or rosemary oil, and a single study showed efficacy against RA [358].

The efficacy of essential oils in anti-obesity trials has been mostly based on in vitro experimental and preclinical studies (Table 4). Essential oils from various plants, flowers, leaves, and roots have been experimentally proven to be effective against obesity based on their anti-inflammatory effects in mice or rats via a common mechanism sharing the pathogenesis of obesity, OA, and RA (Figure 1 and Figure 2). Oral consumption of ginger or garlic oils, inhalation of certain species of lavender oil, or injections of certain citrus essential oils have been shown to result in reductions in body weight, lipid profile, fatty liver disease, and arthritis (Table 4 and references therein).

### 6.3. Medicinal Plants Used to Treat Obesity and Arthritis

Various plant materials produced better results in terms of anti-obesity and anti-inflammatory properties compared to their respective placebo or control groups, as observed in various clinical studies. All of these plants are indispensable parts of different traditional and complementary medicines, and recently, in various randomized clinical trials, they have shown some promising results against obesity and/or arthritis (Table 5). 

Flavonoids are natural polyphenolic compounds with antioxidant, anti-inflammatory, and antiviral properties, as well as protective effects on the gastrointestinal tract [379,380,381,382]. Apigenin, cyanidin, (-)-epigallocatechin-3-O-gallate (EGCG), genistein, kaempferol, luteolin, puerarin, and quercetin are all antioxidants (Table 6); therefore, these compounds demonstrate an inverse relationship between oxidative stress and arthritis, with or without obesity [383,384,385,386,387]. 

Metabolic syndrome (MetS) is a combination of obesity along with high blood pressure and diabetes; obesity can be a driving factor behind the occurrence of MetS. It is said that around one-quarter of the world’s adult population now suffers from MetS. There are conventional drugs for the treatment of obesity, such as orlistat or semaglutide, but these drugs either have adverse effects or are not affordable to the general obese people of low-income countries (LICs) and low–middle-income countries (LMICs) [398]. To reduce obesity, the common, illiterate people, with less means to afford expensive conventional drugs, mostly rely on TMPs, who treat obesity, cardiovascular disorders, and diabetes with medicinal plants. A recent survey lists 16 plants/plant parts used in South Africa for weight loss [398]. These plants include leaves of *Aloe vera* Mill., *Rosmarinus officinalis* L., and *Moringa oleifera* Lam. 

Over 20 plants used to reduce obesity were listed in a review published in 2013; the authors concluded that among the significant anti-obesity plants were *Cissus quandrangularis* L., *Asparagus officinalis* L., and *Zingiber officinale* Roscoe [399]. Another review listed *Curcuma longa* L. rhizomes (active ingredient curcumin) and leaves of *Salvia officinalis* L. (active ingredient: carnosic acid) as anti-obesity plant parts [400]. Obesity as a disorder has been recognized in Ayurveda—the ancient medical treatise of India—where it is described as “meda”. Some Ayurvedic plants/plant parts used to treat obesity in India include the fruits of *Garcinia cambogia* L. (active ingredient: (-)-hydroxycitric acid), *Cyperus rotundus* L. rhizomes (active ingredient: cyperine), the roots of *Embelia ribes* Burm.f., whole plants of *Boerhaavia diffusa* L., seeds of *Achyranthes aspera* L., and roots of *Withania somnifera* (L.) Dunal. [401].

## 7. Conclusions

Some natural anti-obesity agents have been described from dietary sources. These include flavonoids from *Citrus depressa* Hayata, anthocyanins from *Vaccinium ashei* Rehder and *Morus australis* Poir., and gingerol, paradol, and shogaol from *Zingiber officinale* Roscoe [402]. It is evident from several clinical and preclinical trials that essential oils or extracts from aromatic and medicinal plants demonstrate potential therapeutic value against obesity and arthritis (Table 1, Table 2, Table 3, Table 4 and Table 5). These plants and phytochemicals should be considered as functional foods rather than therapeutics, and warrant further extensive clinical studies for dosage and safety determinations for chronic conditions. Importantly, traditional medicines have been used as medicines and foods since prehistoric times. A number of these plant materials (e.g., flavonoids) are used almost every day as a part of our foods, drinks, or spices, and their consumption as medications or therapeutic supplements can help people to avoid the severity of obesity or arthritis (Table 6). The famous Greek physician Hippocrates in 440 BC stated “Let food be thy medicine, and let medicine be thy food”, which is still applicable today. Whether knowingly or unknowingly, human beings do consume at least some bioactive compounds with their daily diet. Traditional medicinal doctors and even scientists recommend that certain foods are beneficial during certain diseases. Although the daily intake of plants containing requisite phytochemicals for a given disorder is also recommended by the authors (Table 1, Table 3, Table 5 and Table 6), we would like to point out that such intake should have scientific evidence behind it, including determination of dosage, frequency of eating, toxicity, and any adverse reactions when taken alone or with other foods. We need to take a closer look at the dietary factors that influence obesity and other inflammatory diseases, obesity and the development of metabolic syndrome, and obesity itself. From this viewpoint, flavonoids such as quercetin, genistein, apigenin, and cyanidin deserve a closer look [403]. 

## Figures and Tables

**Table 1:** Ayurvedic medicinal plants used against arthritis (comparisons of traditional knowledge versus randomized clinical trials). Abbreviations—n: number of patients; VAS: visual analogue scale; KOOS: Knee Injury and Osteoarthritis Outcome Score; PGADA: Patient Global Assessment of Disease Activity; serum sColl2–1: a cartilage degradation marker; SLBSP: solid lipid *Boswellia serrata* particles; BSE: *Boswellia serrata* extract; DAS28: Disease Activity Score-28; WOMAC: Western Ontario and McMaster Universities Osteoarthritis Index; KSF-36: Korean 36-Item Short-Form Health Survey score.

Name	Traditional Use(s)	Clinical Evidence (Total *n*, *n* in Each Group)	Formulation (Treatment Duration, Days)	Arthritis Measurement Parameters	Arthritic Pain Measurement Parameters	Reference
*Curcuma longa*	Against asthma, allergies, food poisoning, rheumatism, liver disorders, and inflammation (rhizome)	Improved RA (total 90, *n* ≅ 30)	2 or 4 caps/day for 84 days (0.25 g/cap turmacin)	Stair mill test	VAS	[152,153]
*Curcuma longa*	-	Decreased knee OA (total 150 *n* ≅ 50)	2 or 3 caps/day for 90 days (46.7 mg turmeric extract)	PGADA; serum sColl2-1	VAS; KOOS	[154]
*Boswellia serrata*	Rheumatism	Decreased knee OA (total 48, *n* ≅ 24)	169.33 mg/cap for 120 days (87.3 mg β-boswellic acids) twice daily	MRI to inspect changes in knee joint gap and osteophytes	Pain and stiffness	[155,156]
*Boswellia serrata*	-	Decreased knee OA (total 43 *n* = 20 (BSE); 23(SLBSP))	SLBSP; BSE: three times daily for 60 days	CTX-II (in urine); IL-2, IL-4, IL-6, TNF-α, and IFN-γ (in serum)	WOMAC, VAS	[157]
*Tinospora cordifolia* (formulation of: *T. cordifolia, Zingiber officinale*, *W. somnifera, and T. terrestris*)	Leprosy, fever, asthma, anorexia, jaundice, gout, skin infections, diabetes, chronic diarrhea, and dysentery	Reduced knee OA (total 121, *n* ≅ 40 per group)	4 caps/day for 168 days (water extracts: 750 mg daily)	Joint counts, global disease assessments, and health assessment questionnaires; plasma inflammatory cytokines.	VAS	[158,159]
*Tinospora cordifolia* (Formulation of: *T. cordifolia, Boswellia serrata, Emblica officinalis, Zingiber officinale*)	-	Reduced knee OA (total 440, *n* = 110 per group)	6 caps for 168 days (2 caps three times daily)	Functional difficulty Likert score	VAS; Modified WOMAC	[160]
***Commiphora myrrha***	Anti-inflammatory, hepatoprotective, muscle relaxing, anti-arthritic, anti-obesity, and anti-malarial	Reduced knee OA (total 100, *n* = 50 per group)	0.5 g (*Commiphora myrrha*) tab twice daily for 84 days	KSF-36, personal evaluation, and laboratory analysis	VAS	[161,162]
*Zingiber officinale*	Colds, nausea, arthritis, migraines, and hypertension	Improved OA (total 60, *n* = 20/group)	Ginger (750 mg cap daily); ginger plus Diclofenac tab (750 mg + 50 mg) for 84 days	WOMAC	VAS	[163,164]
*Zingiber officinale*	-	Improved RA (total 70, *n* = 35/group)	2 caps/day (750 mg ginger/cap) for 84 days	Gene expression of FoxP3, RORγt, and T-bet. Disease Activity Score-28	-	[165]
*Piper nigrum* (mixed with: *Curcuma longa,* and *Zingiber officinale*)		Improved knee OA (total 60, *n* = 30/group) (compared against Naproxen)	2 caps/day for 28 days (ingredients: 300 mg curcumin, 7.5 mg gingerols, and 3.75 mg piperine)	Reduced prostaglandin E2 levels	Beck’s International Questionnaire	[166]

**Table 2:** Recent updates on studies related to plants with anti-arthritic properties used in traditional medicine.

Family	Name	Parts Used	Potential Ingredient(s)	Reference
Acanthaceae	*Andrographis paniculata*	Leaves	Andrographolide	[180,181]
Amaryllidaceae	*Allium sativum*	Essential oil	Diallyl disulfide, diallyl trisulfide, diallyl tetrasulfide	[182]
Anacardiaceae	*Semecarpus anacardium*	Nut, milk extract (as per Siddha formulary)	Bioflavonoids	[183,184,185,186]
Apiaceae	*Centella asiatica*	Leaves (alcoholic extract)	Madecassoside, triterpenoid glycoside, asiaticoside	[187,188,189]
Apiaceae	*Coriandrum sativum*	Herb, fruit, seed, essential oils, hydroalcoholic extract	Cineole	[190,191]
Apocynaceae	*Calotropis procera*	Leaves, seeds, roots	Benzoyllineolone, benzolisolineolone	[192]
Apocynaceae	*Hemidesmus indicus*	Roots	Terpenoids	[193]
Araliaceae	*Acanthopanax chiisanensis*	Leaves	Chiisanoside, chiisanogenin	[194]
Araliaceae	*Panax notoginseng*	Ethanol extract, n-butanol extract	Ginsenoside	[195,196,197,198]
Asparagaceae	*Anemarrhena asphodeloides*	Roots	Mangiferin, polysaccharides, fructan	[199,200]
Asparagaceae	*Asparagus racemosus*	Hydroalcoholic extract	Shatavarin, saponin	[201,202]
Asparagaceae	*Yucca schidigera*	Bark, methanolic extract	Resveratrol, trans-3,3′,5,5′-tetrahydroxy -4′- methoxystilbene, yuccaols, spirobiflavonoids	[203,204,205]
Asteraceae	*Pluchea lanceolata*	Root, hydroalcoholic extract	Sorghumol acetate, boehmerol acetate	[206,207,208]
Asteraceae	*Siegesbeckia orientalis*	Ethanolic extract	Kirenol	[209,210]
Asteraceae	*Tanacetum parthenium*	Inflorescence	Parthenolide	[211,212,213]
Asteraceae	*Tanacetum vulgare*	Aerial parts, methanolic extract, hydroalcoholic extract	3,5-O-dicaffeoylquinic acid (3,5-DCQA)	[214,215]
Asteraceae	*Xanthium strumarium*	Fruits, methanolic extract	Sesquiterpenoids, phenylpropanoids, lignanoids, coumarins, steroids, glycosides, flavonoids, thiazides, anthraquinones, naphthoquinones	[216,217,218]
Berberidaceae	*Berberis vulgaris*	Root extract	Berberine	[219,220]
Boraginaceae	*Arnebia euchroma*	Entire herb (alcoholic extract)	Hydroxy naphthaquinone	[221,222]
Bromeliaceae	*Ananas comosus*	Fruit	Bromelain	[223,224]
Burseraceae	*Boswellia carteri*	Resin	Boswellic acids	[156,225]
Burseraceae	*Boswellia frereana*	Resin	Boswellic acid, epi-lupeol	[226,227]
Burseraceae	*Boswellia serrata*	Resin	3-Oacetyl-11-keto-β-boswellic acid, boswellic acid	[228,229]
Caesalpiniaceae	*Caesalpinia pulcherrima*	Plant, alcoholic extract	ß-Amyrin, glucose, aspartic acid, glycine, proline, caesalpulcherrins	[230,231]
Cannabaceae	*Cannabis sativa Cannabis indica*	Leaves	Cannabidiol	[232,233,234]
Capparaceae	*Capparis spinosa*	Ethanol extract, water extract	P-hydroxy benzoic acid, 5-(hydroxymethyl) furfural; bis(5- formylfurfuryl) ether, daucosterol; α-dfructofuranosides, uracil, stachydrine	[235,236,237]
Caprifoliaceae	*Lonicera japonica*	Dried leaves, dried flowers, water extract	Chlorogenic acid, ioniflavone, polysaccharides	[200,238,239,240,241]
Celastraceae	*Tripterygium wilfordii*	Entire herb, flower, ethyl acetate extracts	Celastrol, macrocyclic dilactone, valerian-type sesquiterpenes, triptolide (diterpene), alkaloids (celabazine, celacinnine, celafurine, and celallocinnine)	[242,243,244,245,246]
Cleomaceae	*Cleome gynandra*	Ethanolic extract	Triterpenes, tannins, anthroquinones, flavonoids, saponins, steroids	[247]
Combretaceae	*Terminalia chebula*	Fruits, hydroalcoholic extract	Chebulic acid, chebulagic acid, chebulinic acid, ellagic acid	[248,249,250,251,252,253]
Convolvulaceae	*Erycibe obtusifolia*	Stems	Scopoletin	[254,255]
Cucurbitaceae	*Citrullus colocynthis*	Herb, aqueous extract	Alkaloids, glycosides, flavonoids, tannins, sterols	[177,256,257]
Cucurbitaceae	*Thladiantha dubia*	Fruit	Polysaccharides	[258]
Cuscutaceae	*Cuscuta reflexa*	Alcoholic extract	Dulcitol, mannitol, sitosterol, lycopene, apigenin-7-β-rutinoside, 6-7 dimethoxy coumarin, quercetin, hyperoside, propenamide, reflexin, lutein, cuscutin, cuscutalin, kaempferol, kaempferol-3-O-glucoside	[259,260,261]
Fabaceae	*Bauhinia tarapotensis*	Leaves (chloroform extract)	Triterpenic acids of ursane and oleanane	[262]
Fabaceae	*Sophora flavescens*	Rhizomes	Kurarinone, kuraridin, isoxanthohumol	[263,264]
Fabaceae	*Trigonella foenum-graecum*	Seeds, alcoholic extract,	Choline, mucilage, trigonelline	[177,265,266,267]
Lamiaceae	*Lavandula multifida*	Aerial parts, essential oils	Linalool, camphene, linalyl acetate, α-thujene, bornyl acetate, β-caryophellene	[262,268]
Lamiaceae	*Leucas aspera*	Ethanolic extract	Epicatechin, β-epicatechin, procyanidin, β-sitosterol	[269,270]
Lamiaceae	*Rosmarinus officinalis*	Aerial parts, water extract, ethanol extract, essential oils	Carnosic acid, α-pinene, camphene, β-pinene, myrcene	[271,272,273,274]
Lamiaceae	*Salvia miltiorrhiza*	Flower, hydroalcoholic extracts	Tanshinone, cryptotanshinone	[275,276,277]
Lamiaceae	*Vitex negundo*	Seeds, leaves,	Lignans (e.g., vitexdoins), Tris(2,4-di-tert-butylphenyl) phosphate	[278,279]
Lauraceae	*Cinnammomum zeylicanium*	Bark, essential oil	Cinnamaldehyde, eugenol, cymene, caryophyllene	[177,178,179]
Lauraceae	*Lindera aggregata*	Dry roots	Norisoboldine	[280,281,282]
Lauraceae	*Litsea guatemalensis*	Etanolic extract, essential oils	5,7,3′,4′-Tetrahydroxy-isoflavone, pinocembrin, scopoletin	[283]
Lecythidaceae	*Barringtonia racemosa*	Fruits	Bartogenic acid	[284]
Loganiaceae	*Strychnos nux-vomica*	Seeds	Brucine, brucine n-oxide, strychnine	[285,286,287]
Lythraceae	*Punica granatum*	Seeds, leaves (juice), methanolic extract	Gallic acid, anthocyanins, ellagic acid, tannins, flavones, flavonoids, anthocyanidins, sterols	[288,289,290,291]
Malvaceae	*Abutilon hirtum*	Herb, essential oil	β-sitosterol, tocopherol, α-pinene, caryophyllene, caryophyllene oxide, endesmol, farnesol, borenol, geraniol, geranyl acetate, elemene and α-cineole	[292,293]
Malvaceae	*Sida rhombifolia*	Aerial parts, stems, roots, hydroalcoholic extract	Flavonoids, tannins, vitamin C	[294,295]
Meliaceae	*Dysoxylum binectariferum*	Seeds	Rohitukine	[296]
Oleaceae	*Olea europaea*	Leaves, fruit, compression-extracted oil	Omega-3 fatty acids, hydroxytyrosol	[297,298,299,300,301]
Oxalidaceae	*Biophytum sensitivum*	Inflorescence	Amentoflavone, polysaccharide	[227,302]
Paeoniaceae	*Paeonia lactiflora*	Flowers, roots,	Glucosides, gallic acid	[303,304]
Phyllanthaceae	*Phyllanthus amarus*	Aqueous extract	Phyllanthin, hypophyllanthin	[305,306,307,308]
Piperaceae	*Piper longum*	Seeds, aqueous extracts	Piperine, piperlongumine, piperlonguminine, methyl 3, 4, 5-trimehoxycinnamate	[309,310,311]
Poaceae	*Saccharum officinarum*	Whole plant, wax oil	Palmitic, oleic, linoleic, and linolenic acids	[312,313]
Polyporaceae	*Poria cocos (saprophytic fungus)*	Sclerotium	Triterpenoids	[314]
Ranunculaceae	*Clematis vitalba*	Aerial parts	Vitalboside	[315]
Ranunculaceae	*Coptidis rhizoma*	Roots and rhizomes	Berberine	[258,316]
Ranunculaceae	*Nigella sativa*	Seeds, compression-extracted oil	Thymoquinone	[317,318,319,320]
Rosaceae	*Chaenomeles speciosa*	Hydroalcoholic extract	Chlorogenic acid	[321,322,323,324]
Rosaceae	*Rosa canina*	Water extract	Terpenoids, galactolipids, carotenoids, fruit acids, fatty oils, phenolics,	[325,326,327]
Rubiaceae	*Lasianthus acuminatissimus*	Roots (methanolic and ethyly acetate extracts)	Anthraquinone glycosides, lasianthuoside, codonolactone	[328,329]
Rutaceae	*Ruta graveolens*	Methanolic extract	8-Methoxycoumarin	[330,331,332]
Solanaceae	*Cestrum diurnum*	Leaves, alcoholic extract	Ursolic acid	[333,334]
Solanaceae	*Withania somnifera*	Roots, leaves, water extract	Withanolides (steroidal lactones)	[335,336,337]
Verbenaceae	*Lantana camara*	Leaves, methanolic extract	Triterpenoids	[338,339,340]
Verbenaceae	*Lawsonia inermis*	Leaves, hydroalcoholic extract	Lawsone, luteolins, apigenin, esculetin, scopletin	[341,342]
Xanthorrhoeaceae	*Aloe vera*	Gel from leaves	Anthroquinone glycosides	[343,344]
Zingiberaceae	*Alpinia officinarum*	Rhizomes	Diaryl heptanoids	[345]
Zingiberaceae	*Curcuma longa*	Rhizome	Curcumin	[346,347,348]
Zingiberaceae	*Zingiber officinale*	Rhizome, alcoholic extract	Gingerols, gingerdiols, phenylpropanoids, [6]-shogaol, shogaols	[349,350,351,352]

**Table 3:** Essential oils used to treat RA in randomized clinical trials (RCTs).

Oil Type	Key Findings	Reference
Evening primrose oil	Patients with RA (*n* = 40 total) and NSAID-induced GI lesions treated with γ-linolenic acid 540 mg/day (evening primrose oil 6 g/day) for 3 months slightly improved RA-related morning stiffness.	[355]
Lavender oil	Aromatherapy with lavender oil improved arthritic pain (against placebo) in patients (*n* = 30 each group) with knee osteoarthritis, but no proof of its long-term efficacy.	[356]
Lavender oil	Aromatherapy with lavender oil improved daily routine activities of patients (*n =* 30 each group) with knee osteoarthritis (against placebo), but no proof of its long-term efficacy.	[357]
Mouthwash with essential oils and curcumin	Gargling with mouthwash containing essential oils and curcumin (MEC) over 6 weeks reduced periodontal disease and RA-related parameters (*n =* 15 each group)	[354]

**Table 4:** Essential oils used to treat obesity and arthritis in preclinical trials.

Essential Oil	Key Findings	Reference
Garlic essential oil	Daily consumption of garlic essential oil (25, 50, and 100 mg/kg) or diallyl disulfide (10 and 20 mg/kg) for 12 weeks in C57BL/6J mice prevented the development of non-alcoholic fatty liver disease. The oil and its major compound also significantly prevented the release of proinflammatory cytokines from murine livers.	[359]
Ginger essential oil	Ginger essential oil (28 mg/kg/day i.p. for 4 weeks) treatment improved joint inflammation caused by streptococcal cell-wall-induced arthritis in female Lewis rats.	[351]
*Pogostemon cablin* Benth. or patchouli essential oil	Inhalation of the oil reduced food intake, systolic blood pressure, and plasma low-density lipoprotein cholesterol levels in SD rats.	[360]
*Rhaponticum acaule* (L.) DC.	Treatment inhibited xanthine oxidase and turkey pancreatic lipase, thus reducing oxidative stress and pancreatitis.	[361]
Ginger essential oil (GEO)	Male C57BL/6J mice with a high-fat diet (HFD) mixed with GEO (12.5, 62.5, and 125 mg/kg) or citral (2.5 and 25 mg/kg) for 12 weeks showed improved HFD-induced obesity by reducing triglyceride and total cholesterol levels. In addition, the treatment reduced inflammatory response in murine liver.	[362]
*Pinus koraiensis* Siebold and Zucc. leaf essential oil	Treatment inhibited the level of cholesterol acyltransferase-1 and -2, as well as low-density lipoprotein (LDL) oxidation activity; thus, it may act against hyperlipidemia.	[363]
*Citrus aurantifolia* (Christm.) swingle essential oil	Forty-five days of treatment with this oil (125 mg/kg/day, s.c.) prevented ketotifen (32 mg/kg/day s.c.)-induced body weight gain and food intake in mice.	[364]
*Artemisia annua* L. essential oil	Treatment reduced obesity-related PPAR-γ, C/EBP-α, SREBP-1c, FAS, and ACC levels in vitro using 3T3-LI cells.	[365]
*Lavandula pubescens* Decne. essential oil	*L. pubescens* EO was assessed against pancreatic lipase inhibitory activity with an IC_50_ of 1.08 μL/mL (in vitro).	[366]

**Table 5:** Clinical trial results of medicinal plants or phytochemicals against obesity.

Material	Key Findings	Reference
*Nigella sativa* oil	Daily consumption of capsules with oil (2 mg/day over a period of 8 weeks) improved HDL-C and lowered LDL-C and TC/HDL-C ratio compared to placebo in obese and overweight women.	[367]
*Nigella sativa* oil	Patients were given a low-calorie diet supplemented with *Nigella sativa* oil (3 g/day for 8 weeks) (*n =* 45 each group), which reduced TNF-α and hepatic C-reactive protein levels, but no changes were observed in plasma IL-6 levels in obese (BMI: 30–35 kg/m^2^) women aged 25–50 years old.	[368]
*Opuntia ficus-indica*	Natural fiber complex (litramine) was 3 g/day with a low-calorie diet for 12 weeks, which reduced body weight compared to placebo in obese women (total *n =* 133)	[369]
*Camellia sinensis*	Green tea (*n =* 32; 1 g of dry green tea extract in capsule/day) reduced total cholesterol (TC) and LDL-C after 12 weeks of treatment in non-diabetic obese women.	[370]
*Crocus sativus*	Saffron reduced hyperglycaemia and hyperlipidaemia and improved liver function in patients with type 2 diabetes in an 8-week randomized clinical trial.	[371]
*Laminaria digitata* (brown seaweed)	Treatment with sodium alginate from *Laminaria digitata* over a period of 10 days showed no effects in an anti-obesity related trial.	[372]
*Lycium barbarum* (fruit juice)	A single-day bolus drink increased metabolic rate; 120 mL of fruit juice per day for 2 weeks reduced waist circumference in overweight men and women (*n =* 15, BMI = 29, age = 34 years).	[373]
*Allium sativum*	Consumption of 1.6 g of garlic powder (4 × 400 mg tablets daily, for 12 weeks) produced significant decreases in waist circumference and body fat percentage in patients with non-alcoholic fatty liver disease (*n =* 45).	[374]
*Allium cepa*	Onion powder (9 g per day for 12 weeks) did not cause any major changes between groups.	[375]
*Persea americana*	Avocados are a natural source of lutein. Daily oral consumption of 300 mg/day of ASU-E (Avocado–Soybean Unsaponifiables, Expanscience—a formula with a 1:3 ratio of avocado: soybean oil) for 3 years did not cause any changes in joint space width loss compared to the placebo group.	[376]
*Momordica charantia*	Oral consumption of *Momordica charantia* (3 × 500 mg per capsule daily for 3 months) taken thrice daily reduced body weight, body mass index, fasting blood glucose levels, and Knee Injury and Osteoarthritis Outcome scores.	[377]
*Cissus quandrangularis*	Consumption of (*n =* 35) aqueous extract of *Cissus quandrangularis* (300 mg/day, over 8 weeks) reduced body fat and improved blood parameters related to metabolic syndrome in overweight patients.	[378]

**Table 6:** Role of flavonoids against obesity and arthritis (preclinical and clinical studies).

Flavonoid’s Name	Role against Obesity or Arthritis	Reference
Apigenin	RA was induced by 0.1 mL Freund’s complete adjuvant (FCA) injections in the palmar surface of paws of Sprague–Dawley (SD) rats. Apigenin suppressed the expressions of P2X7/NF-κB signaling and associated RA-related inflammatory reactions (e.g., reduced IL-1β, Il-6 and TNF-α)	[388]
Apigenin	RA was induced in a murine collagen-induced arthritis (CIA) model. Apigenin inhibited CIA by repressing synovial hyperplasia (by reducing the multiplication of fibroblast-like synoviocytes), causing the growth of new blood vessels and osteoclastogenesis.	[389]
Cyanidin	The effects of cyanidin-3-O-glucoside were investigated in a murine high-fat-diet-induced non-alcoholic fatty liver disease (NAFLD) model. Treatment with this flavone reduced NLRP3 inflammasome activation, oxidative stress, and steatosis in mice.	[390]
(-)-Epigallocatechin-3-O-gallate (EGCG)	Over a period of 3 days, 300 mg of EGCG drink increased postprandial fat oxidation in obese men similarly to 200 mg of caffeine, but the effect was not observed with 600 mg of EGCG drink. Limitation: total *n* = 10, pilot study.	[391]
(-)-Epigallocatechin-3-O-gallate (EGCG)	Consumption of EGCG and resveratrol (282 mg and 80 mg/day over a period of 12-week accordingly) increased oxidative capacity in permeabilized muscle fibers, but showed reduced plasma triacylglycerol concentration in a high-fat mixed-meal assay in obese men (*n* = 18).	[392]
Genistein	Consumption of 15 g of genistein for 3 months (5 days of daily administration per week plus 2 days without treatment) in adult patients (53% men) reduced blood glucose and malondialdehyde levels, but did not impact on lipid profile.	[393]
Kaempferol	Treatment with 200 mg/kg of kaempferol (over eight weeks) with a high-fat diet in C57BL/6 mice reduced the increases in body and liver weight, serum cholesterol, and triglyceride levels	[394]
Luteolin	Luteolin increased the expression of liver X receptor (LXR)-α (in vitro). Luteolin (0.05% *w*/*w* in high fat diet) reduced plasma cholesterol and low- and very-low-density lipoprotein cholesterols in male C57BL/6 mice.	[395]
Puerarin	Obese women with polycystic ovary syndrome (PCOS) took 150 mg/d of puerarin tablets for 3 months in addition to their standard treatment, and showed decreased total cholesterol and systolic blood pressure compared with their pre-treatment levels.	[396]
Quercetin	Quercetin (500 mg/day for 8 weeks) reduced RA symptoms (based on an assessment questionnaire) and high-sensitivity tumor necrosis factor α (hs-TNF-α) in women with RA.	[397]
